# Supplementary material for: Climate Refuges in Nigeria for Oil Palm in Response to Future Climate and Fusarium Wilt Stresses
Source: Plants (Basel). 2023 Feb 8;12(4):764. doi: 10.3390/plants12040764 (PMC9967377; doi:10.3390/plants12040764)
Supplement: Supplementary file 1 [file plants-12-00764-s001.zip › plants-2103858-supplementary.pdf]

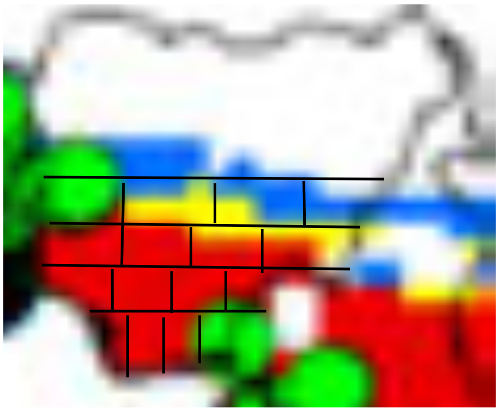

Figure S1. Working document for current time demonstrating the various zones from north north to south south and east east to west wet in the map of Nigeria [23] and in Figure 6. The 2050 maps for Nigeria employed the same zones. The red and yellow zones are highly suitable and suitable climates for growing oil palm and the areas of these were combined to give the combined suitable climate value. The green zones are existing plantations and have at least suitable climate. The 2050 map did not have existing plantations illustrated.
